# A Comparison of the Predictive Validity of Self-Esteem Level and Directly Measured Self-Esteem Stability in the Temporal Prediction of Psychological Distress

**DOI:** 10.3389/fpsyg.2020.01770

**Published:** 2020-07-24

**Authors:** Marcus Roth, Tobias Altmann

**Affiliations:** Department of Psychology, University of Duisburg-Essen, Essen, Germany

**Keywords:** self-esteem stability, validity, psychological distress, longitudinal study, assessment

## Abstract

In contrast to the widely used assessment approach in which self-esteem stability is measured as the standard deviation of repeated measurements, direct measurements of self-esteem stability have hardly ever been implemented in longitudinal studies. The primary goal of the present study was to examine the temporal stability and predictive validity of a direct assessment of self-esteem stability compared with the trait level of self-esteem with respect to the prediction of psychological distress (PD). We examined a sample of 136 employees who completed self-report measures of both self-esteem level [Rosenberg Self-Esteem Scale (RSES)] and self-esteem stability [Self-Esteem Stability Scale (SESS)] along with a measure of PD (SCL-90) at two time points across an interval of 1 year. The results underline the relevance of perceived self-esteem stability in the temporal prediction of PD: After controlling for initial PD, we found that self-esteem stability predicted PD better than self-esteem level did. Therefore, we recommend that the RSES be expanded by adding the three SESS items that directly measure the stability of self-esteem.

## Introduction

### Level of, Changes, and Fluctuations in Self-Esteem

Self-esteem, defined as a global, stable, and affectively loaded index of individual’s attitude or evaluation of the self, is arguably one of the most studied constructs in psychology (for a review, see Donnellan et al., 2011). To measure self-esteem, the Rosenberg Self-Esteem Scale (RSES; Rosenberg, 1965) remains by far the most widely used instrument (Orth et al., 2018). In a systematic review, Gray-Little et al. (1997) concluded that the RSES is a reliable and valid measure of global self-worth and “deserves its widespread use and continued popularity” (p. 450). The RSES comprises five positively and five negatively worded items. The scale was conceptualized as a single-factor scale with scores placed along a continuum that ranges from low to high self-esteem. According to Rosenberg (1979), the individual with a high level of self-esteem can be characterized as follows: “he has self-respect, considers himself a person of worth. Appreciating his own merits, he nonetheless recognizes his faults […] The term ‘low self-esteem’ […] means that the individual lacks respect for himself, considers himself unworthy, inadequate, or otherwise seriously deficient as a person” (p. 54). However, a considerable number of studies applying exploratory or confirmatory factor analysis (CFA) have provided evidence to suggest that the scale consists of two dimensions, which should be interpreted as a methodical artifact of (positive versus negative) item-wording (e.g., Greenberger et al., 2003; DiStefano and Motl, 2006; Roth et al., 2008). Therefore, as recommended by Tomas and Oliver (1999), it might be reasonable to assumed “the existence of a single factor (global self-esteem) underlying responses to Rosenberg’s scale. However, the inclusion of method effects is needed to achieve a good model fit” (p. 94).

As shown by several studies using a longitudinal approach to examine stability and changes in self-esteem in individuals over time (e.g., Kuster and Orth, 2013; Wagner et al., 2016; Shikishima et al., 2018), medium-term stability of self-esteem is relatively large, even across longer periods of time (e.g., 3 years), demonstrating the trait-like characteristic of this construct. However, as has also been shown for other personality traits such as the “Big Five” (e.g., Soto et al., 2011), self-esteem has been found to change in a systematic, normative way over the life span. As recently shown in a comprehensive meta-analysis by Orth et al. (2018) using longitudinal data from 331 independent samples, on average, self-esteem increases in early and middle childhood (from ages 4 to 11), remains constant in adolescence (from ages 11 to 15), increases strongly in young adulthood (until age 30), continues to increase in middle adulthood (until age 60), peaks between 60 and 70, and declines in old age and more strongly in very old age.

Besides the change in trait self-esteem over the life span, self-esteem has also been proposed to change from moment to moment. These barometric or short-term fluctuations (over hours, days, or weeks) within the person are referred to as the stability or instability of self-esteem. Kernis et al. (1989) suggested that self-esteem instability can be conceptualized as the magnitude of short-term fluctuations in global self-esteem. Kernis (2005) defined unstable self-esteem as a dispositional tendency that reflects “fragile, vulnerable feelings of immediate self-worth that are influenced by the vicissitudes of potential self-relevant events” (p. 1575).

To assess the stability of self-esteem, two general approaches can be distinguished (for detail, see Altmann and Roth, 2018): The first approach is a cross-sectional *direct assessment* using a scale on which participants are asked to directly rate any fluctuations in self-esteem they tend to experience in a single measurement occasion. The second approach is an *indirect assessment* in which the standard deviation of multiple individuals’ self-esteem level scores (usually RSES scores) across daily assessments obtained in naturalistic contexts is computed. This approach, which was pioneered by Kernis et al. (1989, 1992) and has also been labeled “statistical” self-esteem instability (e.g., Schubert and Bowker, 2019), has been said to provide the most valid assessment and is hence called the “gold standard” (Chabrol et al., 2006, p. 137) against which direct assessment scales have to be measured. With respect to this, Altmann and Roth (2018) compared the three often applied directs assessment scales—the Instability of Self-Esteem Scale (ISES; Chabrol et al., 2006), an RSES derivate by Kernis et al. (1992), and the Stability of Self-Scale (RSS; Rosenberg, 1965)—with a newly developed scale, consisting of three items only: the Self-Esteem Stability Scale (SESS). As shown by Altmann and Roth (2018), all four measures were substantially correlated with the “gold standard” (i.e., the standard deviation of repeated measures of RSES), and the SESS was its best predictor.

Theoretically, the extent of short-term fluctuations is conceptually distinct from the level of trait self-esteem. Accordingly, Kernis (1993) claims that self-esteem level and self-esteem instability are independent constructs. By contrast, on the basis of sociometer theory by Leary and Baumeister (2000), the two constructs are expected to be connected. According to this theory, individuals with a high level of self-esteem do not lower their state self-esteem easily even when experiencing events that indicate a low relational evaluation because they have a high stable expectation of acceptance. Interestingly, this fact was already anticipated by Rosenberg (1965), who stated, “People with low self-esteem are much more likely than those with high self-esteem to have unstable self-conceptions” (p. 152). Consistent with sociometer theory, empirical studies seem to support these assumptions by showing that stability and level of self-esteem are often correlated. For example, in a meta-analysis of 25 studies that used university samples, Okada (2010) found a weighted mean correlation between self-esteem level and self-esteem instability of -0.31. Because of the weak magnitude of the correlation, Okada (2010) concluded that “treating the two dimensions of self-esteem as independent is not so problematic” (p. 245). However, the results of this meta-analysis were exclusively based on studies that used the indirect assessment method to measure self-esteem instability (i.e., the standard deviation of multiple assessments). Studies using the direct assessment via self-reports of stability found correlations that were often higher, between 0.40 and 0.60 (e.g., van Prooijen, 2016; Tuijl et al., 2018). The circumstance that the direct assessment method of stability was more strongly correlated with the trait level of self-esteem (for a direct comparison of both measurement approaches, see also Schubert and Bowker, 2019) may be a result of the fact that individuals with high self-esteem believe that their own self-esteem is robust and unchanged. In this regard, the stability items of the direct measurement approach would be part of trait self-esteem. Therefore, it is necessary to further examine the dimensionality of self-reported self-esteem level and self-reported self-esteem stability (as measured directly) to decide whether the two constructs are independent.

### Relevance of Self-Esteem Level and Self-Esteem Stability

Until now, the importance of self-esteem as a fundamental construct in psychology has been emphasized by a considerable number of studies that have demonstrated that self-esteem level plays an important role in predicting various kinds of life outcomes such as subjective well-being (e.g., Diener and Diener, 2009), deviant behavior (e.g., Trzesniewski et al., 2006), depression (Orth and Robins, 2013), and health problems (von Soest et al., 2018). It is not surprising that a search of the DSM-IV-TR by O’Brien et al. (2006) showed that “the term ‘self-esteem’ appears in 24 different diagnostic contexts, as a criterion for disorders (e.g., dysthymia), as a criterion for disorders being considered for inclusion in future DSM editions (e.g., depressive personality disorder), and as an associated feature of disorders (e.g., social phobia)” (p. 306).

Although different forms of psychological distress (PD) are often assumed to reflect low self-esteem, some contemporary research studies have argued that the stability of self-esteem is a better predictor of PD than global self-esteem levels are. For example, in a prospective study, Franck et al. (2016) compared the predictive value of self-esteem level with that of self-esteem stability (measured via the variance in multiple assessments) in predicting the development of postnatal depression. After controlling for initial depression symptomatology, they found that self-esteem instability—but not level of self-esteem—significantly predicted postnatal depressive symptoms. Franck et al. (2016) concluded that the temporal fluctuation in self-esteem rather than level of self-esteem appears to be the more important vulnerability factor. In line with this, other studies have also revealed the relevance of self-esteem stability for several constructs such as aggression (e.g., Lee, 2014; Zeigler-Hill et al., 2014), anxiety, depression (Tuijl et al., 2018), psychoses (Murphy et al., 2018), or general psychopathology (e.g., Schiller and Shahar, 2013). Most of these studies used the indirect or statistical approach to measure the stability of self-esteem. This was especially true for studies that longitudinally predicted outcomes from trait level and stability of self-esteem. Until now, to our knowledge, no studies have used a direct approach that goes beyond a cross-sectional design, which is problematic because of the confounding of predictor and criterion and the failure to control for initial symptoms. The only exceptions are studies that have used very short time intervals ranging from 10 days (Chabrol et al., 2006) to 5 weeks (Webster et al., 2017) to calculate the retest reliability of the direct self-reports. Although these studies have revealed good retest stabilities (*r*_*tt*_ = 0.80 or higher), the longitudinal stability of self-reported self-esteem stability has yet to be determined.

### Arguments for Using Direct Measures of Self-Esteem Stability

There are several good reasons to use the direct method in contrast to the indirect statistical procedure, even if the latter approach is considered the “gold standard.” Although the indirect statistical procedure assesses variability in a naturalistic context, it requires participants to invest considerably more time and effort because they have to fill out the RSES repeatedly and with no prompting from the researchers, and then they must return the questionnaires by themselves. These issues might keep researchers from applying this procedure in their studies. Besides these economic reasons, there are also some substantive reasons: First, multiple applications of the same items several times a day (or a week) could lead to fatigue (random responses), consistency (overly similar responses), and reactance (contradictory responses). Second, assessing self-esteem stability with a direct approach improves comparability across studies. Previous studies using the indirect approach have varied in the number of measurements on which the standard deviation was calculated as well as in the period of time during which the assessments were conducted (days, weeks). It is unclear what influence these differences may have had on the scores and whether these scores can be compared (see Altmann and Roth, 2018; Tuijl et al., 2018).

Of course we do not want to conceal the main problem of direct assessment, namely, the risk of memory biases, because direct assessments require participants to reflect on their past experiences, a practice that is prone to memory distortion effects (e.g., Schacter, 1999). However, in our view, because of the flaws mentioned with respect to the indirect or statistical approach, the method of direct assessment deserves further attention.

## Aim of the Current Study

As mentioned above, until now, direct measurements of self-esteem stability have hardly ever been implemented in longitudinal studies. Both the temporal predictive power of the direct or statistical approach and its temporal stability have yet to be determined. Therefore, we extended previous findings by using a research design in which participants completed self-report measures of both self-esteem level and self-esteem instability along with a measure of PD at two time points across an interval of 1 year.

First, we hypothesized that the two aspects of self-esteem—its global level and its stability—represent two distinguishable dimensions. However, on the basis of sociometer theory as well as previous results, we also expected that high self-esteem would be positively related to self-esteem stability. Second, we expected that the stability or instability of self-esteem would be found to be—similar to level of self-esteem—relatively stable across the 1-year assessment interval. Finally, we expected that, when we controlled for initial levels of PD at Time 1, self-esteem level scores as well as stability at Time 1 would make unique contributions to PD scores at Time 2. Comparing the predictive power of the two scores, we expected that self-esteem stability would be the better predictor of PD than the global level of self-esteem. Data and material of the presented study are openly accessible at osf.io/sy59r.

## Materials and Methods

### Data Collection and Procedure

The sample used in the present study had served as the control group in a previous intervention study aimed at evaluating the effectiveness of empathy training in medical caregivers. Three ethics committees approved all aspects of the study: the “Ethikkommission der Abteilung Informatik und Angewandte Kognitionswissenschaft der Fakultät für Ingenieurwissenschaften der Universität Duisburg-Essen” (no reference number given by the committee), the “Ethikkommission der Medizinischen Fakultät der Universität zu Köln” (no reference number given by the committee), and the “Ethikkommission – Medizinische Fakultät Bonn” (reference number: 154/16). Participants were tested four times across an interval of 12 months (without any interim intervention). During the first and last measurement points, self-esteem variables were measured along with PD. Therefore, these two measurement points are referred to in the following as Time 1 and Time 2.

Participants were recruited mainly via flyers and e-mail (staff lists) distributed to the nursing staff at the university hospitals in Essen and Duesseldorf, Germany. The participants received monetary compensation (100 Euros) for their participation, and they were ensured of the confidentiality and anonymity of their data (only code numbers were assigned). Informed consent has been given by all participants in the study. A total of 186 individuals took part in the study (as the control group). At both measurement points, participants completed a questionnaire in groups of eight to 15 people in the presence of an investigator-in-charge. Of the 186 participants, *N* = 136 participants took part in the study until the end.

### Sample

The sample was composed of 136 nurses (18.4% men, 81.6% women) between the ages of 20 and 61 years (*M* = 39.2, *SD* = 11.3) at the first time point. High school diplomas were distributed as follows: 30.1% completed the medium level (completed the 10th grade), and 69.9% completed the highest level (completed the 12th or 13th grades).

### Measures

#### Self-Esteem Level

The German adaptation (Ferring and Filipp, 1996) of the RSES (Rosenberg, 1965) was administered as a measure of an individual’s global level of self-worth. The RSES comprises five positively (e.g., “On the whole, I am satisfied with myself,” “I take a positive attitude toward myself”) and five negatively worded items (e.g., “I feel I do not have much to be proud of,” “I wish I could have more respect for myself”). The scale was conceptualized as a single-factor scale with scores ranging from low to high levels of self-esteem. Subjects are asked to indicate the extent to which the items describe them, using a six-point Likert scale ranging from 1 = “Does not apply to me” to 6 = “Does apply to me.” The internal consistency in the present sample was α = 0.86 at both measurement points.

#### Stability of Self-Esteem

The SESS by Altmann and Roth (2018) described above consists of three items that directly measure fluctuations in self-esteem (“My attitude toward myself is very stable,” “How I estimate my abilities compared with others changes frequently,” “My positive and negative feelings toward myself often blend into each other”). Subjects are asked to indicate the extent to which the items describe them using a six-point Likert scale ranging from 1 = “Does not apply to me” to 6 = “Does apply to me.” The internal consistencies in the present sample were α = 0.67 (Time 1) and 0.69 (Time 2). The items were mixed with the RSES items when presented to participants.

#### Psychological Distress

To assess global PD, 34 items from the German version (Franke, 1995) of the Symptom Checklist (SCL-90-R) by Derogatis (1994) were administered. The items consist of several symptoms (e.g., “Crying easily,” “Headaches”) following the question “how much did you suffer from … in the last seven days?.” Participants were requested to rate how intense they experienced these symptoms on a five-point Likert-scale ranging from 0 (not at all) to 4 (extremely).

As shown in several studies (e.g., Hessel et al., 2001), factor analyses of the 90 SCL items in representative non-clinical samples have suggested that a general factor for measuring the global intensity of PD may be present. Thus, the instrument appears to measure a single global distress factor instead of nine independent symptom subscales. This global factor reflects the general intensity of psychopathological symptoms and could also be labeled “psychological maladjustment.” Therefore, we chose the three scales that best represent the global factor (depression: 13 items; somatization: 12 items; interpersonal sensitivity: nine items). The internal consistencies of the global 34-item scale in the present sample were α = 0.92 at both time points.

## Results

### Descriptive Statistics and Scale Intercorrelations

Table 1 shows the means and standard deviations for the three scales at both measurement points. Table 2 presents the intercorrelations of the scales. As can be seen, the intercorrelations were medium to large, with the smallest coefficients between PD and the RSES or SESS. The 1-year stability of the scales ranged from *r*_*tt*_ = 0.80 (RSES) to *r*_*tt*_ = 0.65 (SESS). Note that the stability or instability of self-esteem seemed to be stable across the time frame used in this study.

**TABLE 1:** Descriptive statistics.

	Time 1		Time 2	
		
	*M*	*SD*	*M*	*SD*
RSES	4.76	0.73	4.63	0.72
SESS	4.33	0.92	4.26	0.88
PD	18.69	14.51	20.08	15.74

*RSES, Rosenberg Self-Esteem Scale; SESS, Self-Esteem Stability Scale; PD, psychological distress.*

**TABLE 2:** Intercorrelation of scores from Time 1 and Time 2.

	Time 1			Time 2		
		
	RSES	SESS	PD	RSES	SESS	PD
**Time 1**						
RSES	–	0.52	–0.57	**0.80**	0.59	–0.53
SESS		–	–0.34	0.55	**0.65**	–0.44
PD			−	–0.44	–0.36	**0.77**
**Time 2**						
RSES				−	0.58	–0.55
SESS					−	–0.51
PD						−

*RSES, Rosenberg Self-Esteem Scale; SESS, Self-Esteem Stability Scale; PD, psychological distress. The bold font indicates stability coefficients. In all cases, *p* < 0.001.*

### Confirmatory Factor Analysis (CFA)

To check whether the RSES and the SESS represent two distinguishable dimensions (instead of one global dimension), two models were tested via CFA using the R-Package “lavaan” (Rosseel, 2012). Model 1 represents the one-dimensional conception of self-esteem and self-stability with all 13 items (10 RSES items, three SESS items) defined as indicators of a single factor. Model 2 depicts the claim that self-esteem level measured by the RSES and self-esteem stability measured by the SESS represent two substantively distinct dimensions with 10 RSES items defined as indicators of self-esteem level and three SESS items as indicators of self-esteem stability. Of course, the two latent variables were allowed to covary. Error covariances were constrained to zero in the models to avoid opportunistic fitting. Because the multivariate normality distribution of the ML estimation method was not met by the data, the robust Satorra-Bentler MLM estimation approach was used. For the CFAs, our sample size of *N* = 136 can be judged as small but adequate according to the benchmarks provided by Wolf et al. (2013) who used Monte Carlo data simulation techniques to evaluate sample size requirements for commonly applied SEMs. The authors recommended a minimum sample size of *N* = 130 for CFAs with three factors, 13 indicators (with high factor loadings), and a power of 1-β > 0.80.

As shown in Table 3, both models demonstrated unsatisfactory fit indices (TLI < 0.90, RMSEA > 0.08). However, a χ^2^ differences test (using the Satorra-Bentler χ^2^ correction factor) revealed that Model 2 was superior (ΔSBχ^2^ = 9.50, *df* = 1, *p* < 0.01). Therefore, we could assume two distinguishable dimensions. Based on previous studies, we must assume that the unsatisfactory fit of the models may be a result of methodological artifacts related to item wording, as mentioned above. Therefore, as recommended by DiStefano and Motl (2006), we tested a model which (in addition to Model 2) consists of a self-esteem factor (all 10 RSES-items), a second factor representing the direction of item wording (the five negative worded RSES-items), and a factor representing the three SESS-items. Correlations between each of the first two substantive factors and the wording factor were assumed to be zero. Expectedly, this model, referred to as Model 3, demonstrated satisfactory fit-indices (TLI = 0.95, RMSEA = 0.06).

**TABLE 3:** Summary of the fit indices for the estimated models using the RSES and the SESS items (*N* = 136).

Model	χ ^2^	*df*	CFI	TLI	RMSEA	RMSEA (90% CI)	SRMR
1	145.249	65	0.86	0.83	0.11	[0.08, 0.13]	0.07
2	127.081	64	0.89	0.87	0.09	[0.07, 0.12]	0.06
3	80.885	59	0.96	0.95	0.06	[0.02, 0.07]	0.06

*CFI, comparative fit index; TLI, Tucker Lewis index (non-normed fit index).*

### Temporal Prediction of Psychological Distress From Self-Esteem Level and Stability of Self-Esteem

To compare the predictive power of the general level of self-esteem as measured by the RSES with the predictive power of self-esteem stability as measured by the SESS, PD (at Time 2) was predicted by two hierarchical regression models (based on variables measured at Time 1 as predictors). In both models, PD at Time 1 was entered in a first step to control for initial PD because the predictors SESS and RSES were highly correlated with PD, and we were interested on the predictive power of the self-esteem variables that goes *beyond* the prediction of PD by itself. Whereas in Model 1, the RSES was entered in the second step followed by the SESS in the third step, in Model 2, the SESS and RSES were entered in the opposite order. This approach allowed us to evaluate the incremental predictive validity of the SESS over the RSES and vice versa. Additionally, in both models, interaction effects between PD, SESS, and RSES were entered as a final step. To determine the appropriateness of sample size to perform these analyses, we conducted an *a priori* power analysis using G^∗^POWER (Faul et al., 2007) for multiple regression models (constituting α = 0.05, 1-β = 0.80, and a medium effect size of *f*^2^ = 0.10). This resulted in a minimum required number of *N* = 64 when six predictors are included in the model.

Table 4 shows that—of course—PD at Time 1 was the best predictor of PD at Time 2. Beyond this auto-prediction, the two self-esteem variables added a significant proportion of variance, if only 4%. Thereby, the SESS showed clear incremental validity over the RSES, whereas the incremental validity of the RSES over the SESS was virtually zero. As also shown in Table 4, adding interactions between the predictors measured at Time 1 did not improve the prediction of PD at Time 2.

**TABLE 4:** Comparisons of the RSES and SESS in the temporal prediction of psychological distress (PD) at Time 2 using multiple regression models (*N* = 136).

Models/steps	β	*t*	*p*	Δ *R*^2^	*F*	*p*
**Model 1**						
Step 1: PD (t1)				0.59	193.45	< 0.001
PD (t1)	0.77	13.91	< 0.001			
Step 2: RSES (t1)				0.01	4.71	< 0.05
PD (t1)	0.69	10.40	< 0.001			
RSES (t1)	–0.14	–2.17	< 0.05			
Step 3: SESS (t1)				0.03	9.26	< 0.05
PD (t1)	0.68	10.51	< 0.001			
RSES (t1)	–0.05	–0.75	*ns*			
SESS (t1)	–0.19	–3.04	< 0.01			
Step 4: Interactions (t1)				0.00	0.73	*ns*
PD (t1)	0.70	9.00	< 0.001			
RSES (t1)	–0.04	–0.60	*ns*			
SESS (t1)	–0.18	–2.79	< 0.01			
PD × RSES (t1)	0.07	0.61	*ns*			
PD × SESS (t1)	–0.01	–0.07	*ns*			
RSES × SESS (t1)	0.09	1.34	*ns*			
**Model 2**						
Step 1: PD (t1)				0.59	193.45	< 0.001
PD (t1)	0.77	13.91	< 0.001			
Step 2: SESS (t1)				0.04	13.74	< 0.001
PD (t1)	0.70	12.48	< 0.001			
SESS (t1)	–0.21	–3.71	< 0.001			
Step 3: RSES (t1)				0.00	0.57	*ns*
PD (t1)	0.68	10.51	< 0.001			
SESS (t1)	–0.19	–3.04	< 0.01			
RSES (t1)	–0.05	–0.75	*ns*			
Step 4: Interactions (t1)				0.00	0.73	*ns*
PD (t1)	0.70	9-00	< 0.001			
SESS (t1)	–0.18	–2.79	< 0.01			
RSES (t1)	–0.04	–0.60	*ns*			
PD × RSES (t1)	0.07	0.61	*ns*			
PD × SESS (t1)	–0.01	–0.07	*ns*			
RSES × SESS (t1)	0.09	1.34	*ns*			

*t1, Time 1; PD, psychological distress; RSES, Rosenberg Self-Esteem Scale; SESS, Self-Esteem Stability Scale.*

However, for the temporal prediction of PD, it may be appropriate to especially consider participants whose PD at Time 1 was not (yet) highly developed. As discussed and empirically demonstrated predominantly in the psychotherapeutic treatment evaluation literature (e.g., Elkin et al., 1995; Baucom et al., 2009) initial severity in the outcome variable can moderate treatment success. Applied to the present study, high PD at Time 1 may render the influence of self-esteem variables ineffective due to insufficient variation in PD between Time 1 and Time 2. Accordingly, a separate analysis of people with comparably low PD at Time 1 may be required first to enable and then to explain changes in the development of PD over the period of 1 year. Therefore, the comparative regression analyses described above were repeated separately for participants with lower PD and for those with higher PD at Time 1 (separated by a median split of PD scores at Time 1). As shown in Table 5, for participants with lower PD scores at Time 1, stability of self-esteem was a substantially better predictor of PD 1 year later. Again, the SESS showed clear incremental validity over the RSES, whereas the incremental validity of the RSES over the SESS was relatively small. By contrast, for people who already had high PD at Time 1, the predictive power of both self-esteem variables was much lower and only noteworthy for the stability scale.

**TABLE 5:** Comparisons of the RSES and SESS in the temporal prediction of psychological distress (PD) at Time 2 using multiple regression models, separated into lower versus higher PD at Time 1.

	Lower PD (*n* = 68)			Higher PD (*n* = 68)		
		
Models/steps	Δ *R*^2^	*F*	*p*	Δ *R*^2^	*F*	*P*
**Model 1**						
Step 1: PD (Time 1)	0.17	13.42	< 0.001	0.51	67.16	< 0.001
Step 2: RSES (Time 1)	0.10	8.63	< 0.01	0.01	0.71	*ns*
Step 3: SESS (Time 1)	0.05	5.07	< 0.05	0.03	3.55	*ns*
**Model 2**						
Step 1: PD (Time 1)	0.17	13.42	< 0.001	0.51	67.16	< 0.001
Step 2: SESS (Time 1)	0.13	12.32	< 0.001	0.03	4.32	< 0.05
Step 3: RSES (Time 1)	0.02	1.77	*ns*	0.00	0.31	*ns*

*RSES, Rosenberg Self-Esteem Scale; SESS, Self-Esteem Stability Scale.*

## Discussion

Contemporary research has established that fluctuations in self-esteem are more predictive of negative adjustment outcomes than the global level of self-esteem is. However, most of the studies that have used a longitudinal design have applied an indirect approach for the assessment of self-esteem stability, whereas no studies have applied direct assessments of stability to self-reported longitudinal data. Because the direct approach also offers some advantages over the indirect or statistical approach (see above), we tried to bridge this gap in the current study by applying measures of both self-esteem level and self-esteem instability along with a measure of PD at two time points across an interval of 1 year. Furthermore, the majority of studies that have assessed self-esteem stability have usually been based on samples of university students (see also Okada, 2010). We transcended this limitation by using a sample of adult employees.

Consistent with findings from prior studies that have used direct measurements of self-esteem stability and also in line with sociometer theory (Leary and Baumeister, 2000), our study showed that the direct measurement of stability (SESS) and the global level of self-esteem (RSES) are highly correlated. Thus, there is a clear tendency for individuals with high levels of self-esteem to describe their self-esteem as stable—and vice versa. However, as shown by the CFA, self-reported level and stability are two distinguishable dimensions. Of course, the fit indices of the two-factor model are far from perfect, but they are superior to the model with only one global factor. Further, the insufficient fit of the two-factor model has been confirmed to be a result of the item wording of the RSES itself, as shown by a subsequent model test in the present study, in which wording effects of the RSES-items were modeled as a separate factor (see also Tomas and Oliver, 1999; Greenberger et al., 2003; DiStefano and Motl, 2006; Roth et al., 2008).

As also shown by our results, the SESS showed a 1-year stability of *r* = 0.65, which suggests that the scale likely measures a trait-like construct. Thus, the stability or instability itself is a stable characteristic.

As hypothesized, when we controlled for PD scores at Time 1, the RSES and SESS scores each uniquely predicted PD scores at Time 2. As shown by head-to-head comparisons in which the SESS and the RSES where entered into hierarchical regression models in two different orders, the stability of self-esteem as a predictor of PD was superior to the level of self-esteem. This was especially true when PD was temporally predicted in participants whose PD was only low to moderate at Time 1. In this case, only the stability scale predicted later PD (explaining 13% of the variance), whereas no significant improvement could be shown by the global level of self-esteem.

Although our study adds to and extends previous research on the direct assessment of self-esteem stability, several limitations must be kept in mind when interpreting the findings. First, the non-experimental design of our study precludes us from forming any cause-and-effect inferences about the relations between stability, level of self-esteem, and PD. Second, our sample size was comparatively small and the sample of (mostly female) medical care providers constrains the extent to which our findings can be generalized to a broader population (but not any more than the university samples that have commonly been used in previous studies). Furthermore, our assessment interval was 1 year, which seems to be a small range for a longitudinal analysis of the development of PD. Therefore, future research would do well to use larger and more representative samples and to extend the assessment interval. Because we have implemented only one predicted variable (PD) in our study, we cannot conclude whether self-esteem stability is generally superior as a predictor compared with self-esteem level. Our findings are primarily limited to the global measure of PD we used in the present study. Therefore, future research should also extend the bandwidth of predicted variables. Finally, we relied exclusively on self-report measures to operationalize the key constructs in our study. Although the instruments we used demonstrated acceptable consistencies (as well as retest reliabilities over 1 year), like all self-report measures, they are susceptible to distortion because of response styles (e.g., social desirability). With respect to the instrument we used, additional limitations of our study exist in the circumstance that the predictor variables (self-esteem level and stability of self-esteem) were measured with only one scale. Accordingly, we cannot infer whether the two concepts in general or only the inventories used in this study specifically differ in their predictive validity. Therefore, it seems reasonable in future research to measure both traits using a multimethod approach—in the sense of a multitrait-multimethod matrix proposed by Campbell and Fiske (1959).

## Conclusion

Because the SESS consists of only three items, the improvement it offers in prediction over the 10-item RSES, as shown in our study, is considerable. Therefore, we recommend that the standard application of the RSES be expanded by adding the three SESS items in order to measure *both* constructs, the level of self-esteem as well as the stability of self-esteem.

## Data Availability Statement

Data and material of the presented study are openly accessible at osf.io/sy59r.

## Ethics Statement

The studies involving human participants were reviewed and approved by Ethikkommission der Medizinischen Fakultät der Universität zu Köln. The patients/participants provided their written informed consent to participate in this study.

## Author Contributions

TA and MR made substantial contributions to the conception or design of the work, the acquisition, analysis, and interpretation of data for the work, drafting the work, and revising it critically for important intellectual content, final approval of the version to be published, and agreed to be accountable for all aspects of the work in ensuring that questions related to the accuracy or integrity of any part of the work are appropriately investigated and resolved. Both authors contributed to the article and approved the submitted version.

## Conflict of Interest

The authors declare that the research was conducted in the absence of any commercial or financial relationships that could be construed as a potential conflict of interest.
